# Notices

**Published:** 1866-05

**Authors:** 


### NOTICES.

To Subscribers.—We will cheerfully supply subscribers with the numbers which they do not receive, provided application is made during the month for which the missing number was issued, otherwise ten cents must be sent for any back number; the reason for this is that sometimes the publisher is applied to for the back numbers for several months, when nine times out of ten it is the subscriber's fault in not giving specific and plain directions.

All subscriptions must begin with the January number, as from January to December makes up the volume for the year.

To Publishers.—The following is a sample of hundreds of similar letters: "March 16th, 1866. I have been endeavoring for some time past to find where and by whom 'Hall's Journal of Health' was published, but am still none the wiser for my researches, and I write this note, thinking perhaps you are the publisher. If so, send me a number of your Journal. I want to subscribe for the same, and think a number of my neighbors would like to take it." Not long since a gentleman wrote that he just ascertained by accident that the Journal was published in New York city and that it had been upon his mind to take it for six or seven years. Not long ago an inquiry was made as to where that most excellent paper was published, "The Christian Watchman and Reflector." Those papers which have the name of the place of issue as part of their title have a considerable advantage, such as the New York Observer. There are three remedies: let publishers advertise more; in copying from each other, let them state the place of publication; this we have aimed to do for several years; or let the periodicals at least, devote a page now and then to the time, character and price of their exchanges; we did this several years, to the great help of others, but not a penny to ourself, yet it was pleasant to think we were helping others, both subscribers and brother editors; for the same reason we have sent literally thousands of newspapers and magazines to country cousins and others, post paid, when we knew the number was a good one, thinking it might bring a subscriber; and we have very many times wondered if any exchange ever did in a single instance do the same by us. But whether done or not we shall pursue the old plan, for it is a good investment to have acted in such a way as to have the consciousness of trying to help somebody. Make a note of this, brothers of the quill.

COFFEE AND TEA are among the good things of this life when the use of them is not abused. By the ordinary means of preparation a portion of the flavor escapes and is lost, this is effectually retained by the Eureka Coffee and Tea Pot sold by George B. Morse, General Agent, 389 Broadway; it is simple, cheap, convenient and economical.

WEST VIRGINIA.—Its Farms, Forests, Mines and Oil Wells with a glimpse of its scenery, a photograph of its population and an exhibition of its industrial statistics, by J. R. Dodge of the U. S. Department of Agriculture, 276 pp., 12mo., published by J. B. Lippincot & Co. of Philadelphia, with a copious index. This admirably written volume will repay any reader for the time spent in its perusal and to men of intelligence and enterprise who are inclined to develope the resources of this rich domain, the book is invaluable; the volume beautifully closes thus: "with the added influence of churches and schools, rendering the moral atmosphere as pure as the physical, and making the waters of life as pure as the perennial springs of the everlasting hills, the homes of West Virginia may equal in attraction the most favored upon this continent."

Wilson's Presbyterian Historical Almanac, vol. seven, containing the annual chronicles of the Presbyterian Church, will soon be issued. To subscribers who send the money with their names $2.00. To those who prefer to pay on receiving it $2.25. After the Almanac is published the price to all will be $2.50. This enterprise, for securing the materials of the history of the Presbyterian Church in permanent form has met with the highest commendation of the religious press while the most eminent ministers in the church, Professors in Theological Seminaries and the educated laity, have extended to it their hearty encouragement and patronage; it well deserves a place in the library of every intelligent Presbyterian family. Address, Joseph M. Wilson, Philadelphia, Penn

The American Tract Society, 150 Nassau street, have issued "Green Pastures for Christ's Little Ones," which is beautifully instructive and encouraging to the young who are "looking unto Jesus;" 182 pp. 16 mo. "Bertha Alston," or the good stepmother, a narration of great interest, abounding in the inculcation of Christian duties in various stations of domestic life. "Besie," or Honest Industry. Every boy will revel in the reading of this suggestive little volume, and will arise from its perusal with strong resolves against idleness and all wrong doing, and if made in the strength of Him who so much loved little children when he tabernacled among men, a long and happy and useful life will be the pretty sure result.

Air Purifier, by A. S. Lyman, 212 2nd av., N.;Y. city. This is an apparatus for purifying the air of rooms and sleeping apartments; it is placed at the head of the bed, increasing its length but thirteen inches, and is so made as to seem to be an ornamental part of the bed; it certainly accomplishes two things. 1st. It purifies the air. 2nd. It reduces the temperature of a room when required twenty degrees, at an expense of two cents per hour: when understood, the apparatus is simple, requires but little trouble, which bears no comparison to the comfort given, in reducing the temperature of a chamber twenty degrees on a June night, and giving to fevered patients a cool and pure air to breathe, which does more than all medicines to promote the convalescence of the sick from any disease.

Farm House Milk, with all the cream, pure and sweet, is furnished daily at 12th street, near Broadway, and at corner of 37th street and Broadway, by the Rockland county and New Jersey Milk Association, under the vigilant superintendence of J. S. Canfield, Esq. It is the purest milk ever served by milkmen in the city of New York. Persons who are changing their residences cannot do better than to patronize this company.

The article on Ventilation is in reference to a new mode by I. Pitman, Esq. of Providence, Rhode Island.

